# Recent decline in hepatitis E virus prevalence among wild boars in Japan: Probably due to countermeasures implemented in response to outbreaks of classical swine fever virus infection^☆^

**DOI:** 10.1016/j.virusres.2024.199438

**Published:** 2024-07-19

**Authors:** Masaharu Takahashi, Tsutomu Nishizawa, Akira Nishizono, Manri Kawakami, Yukihiro Sato, Kazunori Kawakami, Masahiko Irokawa, Tomoko Tamaru, Shinichi Miyazaki, Mizuho Shimada, Hideaki Ozaki, Putu Prathiwi Primadharsini, Shigeo Nagashima, Kazumoto Murata, Hiroaki Okamoto

**Affiliations:** a Division of Virology, Department of Infection and Immunity, Jichi Medical University School of Medicine, Shimotsuke, Tochigi 329-0498, Japan; b Department of Microbiology, Faculty of Medicine and Research Center for Global and Local Infectious Diseases, Oita University, Yufu, Oita 879-5593, Japan; c Center for Liver Disease, Okayama Saiseikai General Hospital, Okayama, Okayama 700-8511, Japan; d Department of Internal Medicine, Kamiichi General Hospital, Nakaniikawa-gun, Toyama 930-0391, Japan; e Ayagawa National Health Insurance Sue Hospital, Ayauta-gun, Kagawa 761-2103, Japan; f Irokawa Clinic, Hanishina-gun, Nagano 389-0601, Japan; g Nishiizu Ken-ikukai Hospital, Kamo-gun, Shizuoka 410-3514, Japan; h Department of Gastroenterology, Tottori Seikyo Hospital, Tottori, Tottori 680-0833, Japan; i Health Care Center, Jichi Medical University Hospital, Shimotsuke, Tochigi 329-0434, Japan; j Ozaki Homecare Clinic, Yufu, Oita 879-5434, Japan

**Keywords:** Classical swine fever, Genotype, Hepatitis E virus, Prevalence, Wild boar

## Abstract

Previous studies have emphasized the necessity of surveillance and control measures for hepatitis E virus (HEV) infection in wild boars, an important reservoir of HEV. To assess the current situation of HEV infection in wild boars in Japan, this study investigated the prevalence and genetic diversity of HEV among wild boars captured in 16 prefectures of Japan during 2018–2023. Serum samples from 968 wild boars were examined for anti-HEV IgG antibodies and HEV RNA. The prevalence of anti-HEV IgG varied geographically from 0 % to 35.0 %. HEV RNA was detected in 3.6 % of boars, with prevalence varying by prefecture from 0 % to 22.2 %. Genotype 3 was the most prevalent genotype (91.9 %), followed by genotype 4 (5.4 %), with one strain closely related to genotype 6. The prevalence of HEV infection among wild boars decreased from 2018/2019 to 2022/2023 with significant declines in levels of anti-HEV IgG antibodies (14.5 % vs. 6.2 %, *P* < 0.0001) and HEV RNA (7.6 % vs. 1.5 %, *P* < 0.0001). Regional analysis showed varying trends, with no HEV RNA-positive boars found in several regions in recent years. A plausible factor contributing to the decline in HEV infection is the application of countermeasures, including installing fences to prevent intrusion into pig farms, implemented in response to the emergence of classical swine fever virus (CSFV) infection in wild boars and domestic pigs, with incidents reported annually since 2018. Further investigation is warranted to explore the association between countermeasures to CSFV infection and the decrease in HEV infection among wild boars.

## Highlights

• HEV prevalence and genetic diversity were studied in 16 prefectures from 2018-2023.
• Anti-HEV IgG was found in 10.7 % of boars; HEV RNA was found in 3.6 % of boars.
• HEV-3 was most common (91.9 %), with HEV-4 and a HEV-6-related strain also present.
• HEV prevalence in wild boars decreased significantly from 2018/2019 to 2022/2023.
• Decline in HEV among boars may be linked to classical swine fever virus outbreaks.

## 1 Introduction

The hepatitis E virus (HEV) remains a significant global public health concern as a primary cause of acute viral hepatitis. In developing countries, HEV exhibits endemic characteristics, often causing substantial outbreaks, primarily due to inadequate sanitation and contaminated potable water sources. Conversely, in industrialized countries, hepatitis E typically occurs sporadically and in localized clusters and is largely associated with zoonotic transmission pathways (Dalton et al., 2014; Nelson et al., 2019; Purcell and Emerson, 2008). Various domestic and wild animals, including swine, wild boars, deer, mongooses, and rabbits, act as natural hosts for zoonotic strains of HEV. These animal reservoirs are potential sources for human infection as shown by the detection of infectious HEV in commercially available meat and dairy products (Cossaboom et al., 2016; Primadharsini et al., 2021; Takahashi and Okamoto, 2014; Tei et al., 2003; Yazaki et al., 2003). Although most HEV infections are subclinical or only cause mild symptoms, HEV can progress to a chronic condition in immunosuppressed patients (Ma et al., 2022) and may also cause fulminant hepatic failure (Suzuki et al., 2002). Mortality rates range from 0.5 % to 4.0 % in the general population but are markedly higher in pregnant women, patients with pre-existing liver disease, and immunocompromised individuals (Qiu et al., 2023). Growing evidence also indicates that HEV infection is associated with extrahepatic manifestations, including neurological and renal disorders (Pischke et al., 2017).

HEV is a single-stranded, positive-sense RNA virus that circulates in the blood as a membrane-cloaked quasi-enveloped virus but is excreted in the feces as a naked, nonenveloped virion (Nagashima et al., 2017). The HEV genome is approximately 7.2 kilobases (kb) in size and comprises a short 5ʹ-untranslated region (5ʹ-UTR), three open reading frames (ORF1, ORF2, and ORF3), and a short 3ʹ-UTR terminated by a poly (A) sequence (Kabrane-Lazizi et al., 1999; Tam et al., 1991). ORF1 encodes nonstructural proteins involved in replication, ORF2 codes for a capsid protein and glycosylated secretory protein, and ORF3 encodes a small phosphoprotein required for virion egress (Kenney and Meng, 2019; Koonin et al., 1992; Primadharsini et al., 2021).

HEV is classified in the family *Hepeviridae,* which comprises two subfamilies: *Orthohepevirinae* and *Parahepevirinae.* The *Orthohepevirinae* encompasses at least four genera: *Paslahepevirus, Rocahepevirus, Chirohepevirus*, and *Avihepevirus* (Purdy et al., 2022). The genus *Paslahepevirus* contains two species, *P. alci* and *P. balayani*, with the *P. balayani* comprising eight viral genotypes, HEV-1 to HEV-8 (Smith et al., 2020). HEV-1 and HEV-2 strains are exclusively isolated from humans and cause large outbreaks of human hepatitis E, whereas HEV-3 and HEV-4 strains have been isolated from domestic pigs and wild boars and are responsible for sporadic cases of hepatitis E in both developing and industrialized countries (de Deus et al., 2008; Pavio et al., 2010; Wang and Meng, 2021); HEV-5 and HEV-6 strains have only been detected in wild boar populations in Japan (Takahashi et al., 2010; Takahashi et al., 2014; Takahashi et al., 2011; Takahashi et al., 2020); HEV-7 infects dromedary camels (Woo et al., 2014); and HEV-8 infects Bactrian camels (Nishizawa et al., 2021; Woo et al., 2016). HEV-3 is further subdivided into at least 14 subtypes (3a−3m and 3ra), and HEV-4 is subdivided into at least nine subtypes (4a−4i) (Smith et al., 2020). Notably, HEV strains of subtypes 3a, 3b, and 3e, as well as those of subtypes 4c, 4g, and 4i, are circulating and associated with zoonotic food-borne transmission in Japan (Hara et al., 2014; Nakano et al., 2013; Okamoto, 2007; Sato et al., 2011; Takahashi et al., 2020).

Multiple studies have corroborated the susceptibility of various animal species to HEV. Notably, HEV transmission to humans, resulting in hepatitis E, has been documented in swine, wild boars, rabbits, camels, and rats (Abravanel et al., 2017; Lee et al., 2016; Li et al., 2005; Sridhar et al., 2021; Yazaki et al., 2003). Detection of HEV antibodies and RNA in regard to HEV transmission from wild boars has been reported from numerous countries (Fanelli et al., 2021), with documented instances of transmission to humans, particularly in Japan (Li et al., 2005; Matsuda et al., 2003; Okano et al., 2014; Tamada et al., 2004).

Our previous investigations conducted during 2003−2019 consistently revealed the stable prevalence of HEV among wild boars in Japan. The annual seroprevalence rate ranged from 5.0 % to 15.3 % (mean, 9.3 %), with an annual detection rate of HEV RNA ranging from 0.7 % to 7.5 % (mean, 3.8 %) (Nishizawa et al., 2005; Sato et al., 2011; Sonoda et al., 2004; Takahashi et al., 2014; Takahashi et al., 2011; Takahashi et al., 2020). However, recent surveys on HEV prevalence among wild boars in Japan indicate a declining trend due to an unknown etiology. Consequently, to elucidate the current prevalence and genotype/subtype distribution of HEV infection among wild boars in Japan, stratified by year of capture and geographic region (prefecture and hunting area), we conducted serological and molecular analyses of serum samples and/or liver/gall bladder specimens obtained from 1017 wild boars in 16 prefectures in Japan. In addition, we attempted to identify potential factor(s) that contribute to the decline in HEV prevalence among wild boars in Japan.

## 2 Materials and methods

### 2.1 Serum, liver, and gall bladder samples from wild boars

**Fig. 1:**
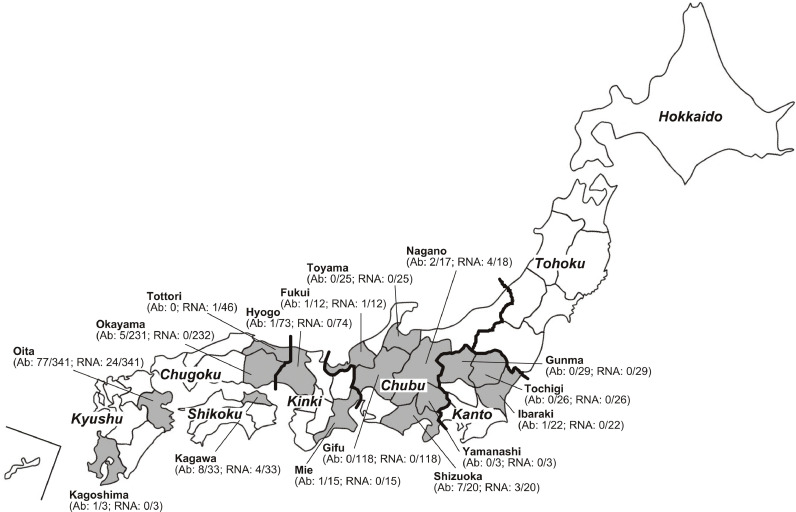
A map of Japan showing eight distinct regions (italicized), including those in mainland Honshu separated by thick lines, and 16 prefectures (outlined as shaded areas) where wild boars were captured. The positive/total number of anti-HEV IgG antibodies and HEV RNA in wild boars captured during the current study is indicated in parenthesis (denoted as Ab and RNA, respectively).

A total of 1017 wild boars (*Sus scrofa leucomystax*) were captured between April 2018 and December 2023 across 16 prefectures spanning north to south in Japan; 968 serum samples, 656 liver tissues, and 46 gall bladder samples were obtained from these samples. Sampled prefectures included Ibaraki (n = 22), Tochigi (n = 26), and Gunma (n = 29) in the Kanto region; Toyama (n = 25), Fukui (n = 12), Yamanashi (n = 3), Nagano (n = 18), Gifu (n = 118), and Shizuoka (n = 20) in the Chubu region; Mie (n = 15) and Hyogo (n = 74) in the Kinki region; Tottori (n = 46) and Okayama (n = 232) in the Chugoku region; Kagawa (n = 33) in the Shikoku region; and Oita (n = 341) and Kagoshima (n = 3) in the Kyushu region of Japan (Fig. 1). Specifically, paired serum and liver specimens were available from 653 boars, serum only from 315 boars, liver tissues only from 3 boars, and gall bladder samples only from 46 boars.

All wild boars were captured by hunters as part of a noxious animal extermination program authorized by the local government. Blood samples were obtained by aspirating cardiac blood using a disposable syringe, and 10 ml were placed in 15 ml screw-capped centrifuge tubes (AS ONE corp., Osaka, Japan). Liver samples were excised into thumb-sized pieces and placed in 50 ml screw-capped conical centrifuge tubes (Thermo Fisher Scientific Inc., Waltham, MA, USA). In addition, gall bladder samples were excised at the cystic duct, which was tied off with string, and stored in plastic bags. The collected samples were transported under refrigerated conditions to the hunters’ residences and immediately stored in a -20°C freezer, although the blood samples became hemolyzed. Subsequently, the samples were transferred to our laboratory in a frozen state. The plasma samples were separated by centrifugation of hemolyzed blood samples at 2100 × *g* for 10 min and stored, along with liver and gall bladder samples, at -80°C until further analysis.

Notably, no overlap was found in the tested wild boars between the current study and previous investigations conducted from January 2003 to December 2019 (Nishizawa et al., 2005; Sato et al., 2011; Sonoda et al., 2004; Takahashi et al., 2014; Takahashi et al., 2020), although an overlap was present in the sampling years of 2018 and 2019.

### 2.2 Detection of anti-HEV antibodies

An enzyme-linked immunosorbent assay was used to identify anti-HEV IgG antibodies in 100-fold diluted plasma samples from wild boars. This assay used the purified recombinant ORF2 protein (rHEV-4 ORF2, amino acids 112–660) from the HE-J1 strain (HEV-4), which was expressed in silkworm pupae, as desribed by Mizuo et al. (2002). The assay procedure followed the protocol established by Takahashi et al. (2005). The positivity criterion for swine anti-HEV IgG was determined by an optical density (OD) value of 0.274 (mean plus six standard deviations), based on the analysis of control swine serum samples (n = 118) that were negative for HEV RNA, sourced from an HEV-free farm (Takahashi et al., 2005). Plasma samples with OD values equal to or exceeding this cut-off value were considered indicative of anti-HEV IgG positivity. To confirm the specificity of the anti-HEV assay, the results were validated through absorption experiments using the same recombinant ORF2 protein that was employed as the antigen probe (Takahashi et al., 2005).

### 2.3 Detection of HEV RNA

To detect HEV RNA, reverse transcription (RT)-polymerase chain reaction (PCR) was performed. Total RNA was extracted from either 100 μl of plasma samples or 100 μl of 10 % (w/v) bile suspensions obtained from gall bladders using two different RNA extraction reagents to minimize PCR inhibitors: the Roche High Pure Viral RNA Kit (NIPPON Genetics Co. Ltd., Tokyo, Japan) and TRIzol LS Reagent (Thermo Fisher Scientific Inc.). For liver specimens, total RNA was extracted from 50 mg of tissue using TRIzol Reagent (Thermo Fisher Scientific Inc.). The bile suspension was prepared by disintegrating a portion of dried or semi-dried gall bladder containing bile (0.4−15.3 g; mean 4.9 g) with a surgical blade, followed by mixing in phosphate-buffered saline (0.1 M sodium phosphate and 0.15 M sodium chloride, pH 7.2, Thermo Fisher Scientific Inc.) at 4 °C overnight. The mixture was then centrifuged at 2100 × *g* for 10 min. The extracted RNA was subsequently used for RT with SuperScript IV reverse transcriptase (Thermo Fisher Scientific Inc.).

Nested PCR (ORF2/3-138 PCR) was carried out using 17-mer or 20-mer degenerate primers (HE361, sense primer for 1st round PCR; HE1114, antisense primer for cDNA synthesis and 1st round PCR; HE830, sense primer for 2nd round PCR; and HE1113, antisense primer for 2nd round PCR, respectively), broadly targeting the well-conserved ORF2/3 overlapping region. The PCR was performed with TaKaRa Ex Taq (TaKaRa Bio, Shiga, Japan) as previously described (Nishizawa et al., 2021). The first and second rounds of PCR yielded amplification products of 176- and 138-base pairs (bp), respectively. Supplementary Fig. S1 demonstrates that the ORF2/3-138 PCR assay can detect all eight genotypes of HEV strains, as verified *in silico* using 53 representative sequences from HEV-1 to HEV-8 (Smith et al., 2020).

For HEV genotyping, another RT-PCR assay targeting the ORF2 region (ORF2-457 PCR) (Mizuo et al., 2002) was performed on ORF2/3-138 PCR-positive samples. The first- and second-round PCR in this case yielded 506- and 457-bp amplification products. The specificity of the RT-PCR assays was verified through sequence analysis, as described below, and the sensitivity of the RT-PCR assays was assessed as previously described (Inoue et al., 2006).

### 2.4 Amplification of the full-length HEV genome

To elucidate the complete genomic sequences of wbJSO_20-1 and wbJSO_20-3 genomes ascertained in the current investigation, total RNA was extracted from 1 mL of serum collected from wbJSO_20-1 and wbJSO_20-3 boars. cDNA synthesis was conducted, followed by nested PCR encompassing five overlapping regions, including both the 5ʹ and 3ʹ termini. Enzymes including KOD Multi & Epi (Toyobo, Osaka, Japan) and TaKaRa LA Taq with GC Buffer (TaKaRa Bio) were employed, along with primers designed using well-conserved regions shared among all HEV strains of genotypes 1−8, for which entire genomic sequences are available. Primer sequences used were derived from sequences obtained during the amplification procedure, in accordance with a previously established method (Takahashi et al., 2014).

Amplified regions, excluding the primer sequences, spanned nucleotide (nt) 1−83 (83 nt), nt 44−2137 (2094 nt), nt 2092−4180 (2089 nt), nt 3953−6335 (2383 nt), and nt 6040−7239 (1200 nt) for both HEV strains. The extreme 5ʹ-end sequence (nt 1−83) was determined using RNA ligase-mediated rapid amplification of cDNA ends technique (RLM-RACE) method, employing the First Choice RLM-RACE kit (Thermo Fisher Scientific Inc.) as described previously (Okamoto et al., 2001). Amplification of the 3ʹ-end sequence for nt 6040−7239 (1200 nt), excluding the poly (A) tail, was conducted using the RACE method, as previously outlined (Okamoto et al., 2001).

### 2.5 The determination and analysis of nucleotide sequence

The amplification product was purified using a FastGene Gel/PCR Extraction Kit (NIPPON Genetics, Ltd., Tokyo, Japan). Subsequently, both strands were directly sequenced or cloned into the T-Vector, pMD20 (TaKaRa Bio), followed by sequencing using an Applied Biosystems 3130xl Genetic Analyzer (Thermo Fisher Scientific Inc.) along with the use of the BigDye Terminator v3.1 Cycle Sequencing Kit (Thermo Fisher Scientific Inc.). The sequence analysis was conducted employing Genetyx (version 13.0.1; Genetyx Corp., Tokyo, Japan), and multiple alignments were generated using MUSCLE, version 3.5 (Edgar, 2004).

Phylogenetic trees were constructed based on the 412-nt ORF2 sequence (nt 5944−6355: accession number M73218) or the full-length sequence, according to the neighbor-joining tree of Jukes-Cantor distances, as implemented in MEGA11 (version 11.0.13) (Tamura et al., 2021). The robustness of the clusters was assessed by performing 1000 bootstrap replicates, and branches with bootstrap values >70 % were grouped together (Tamura et al., 2021).

**Fig. 2:**
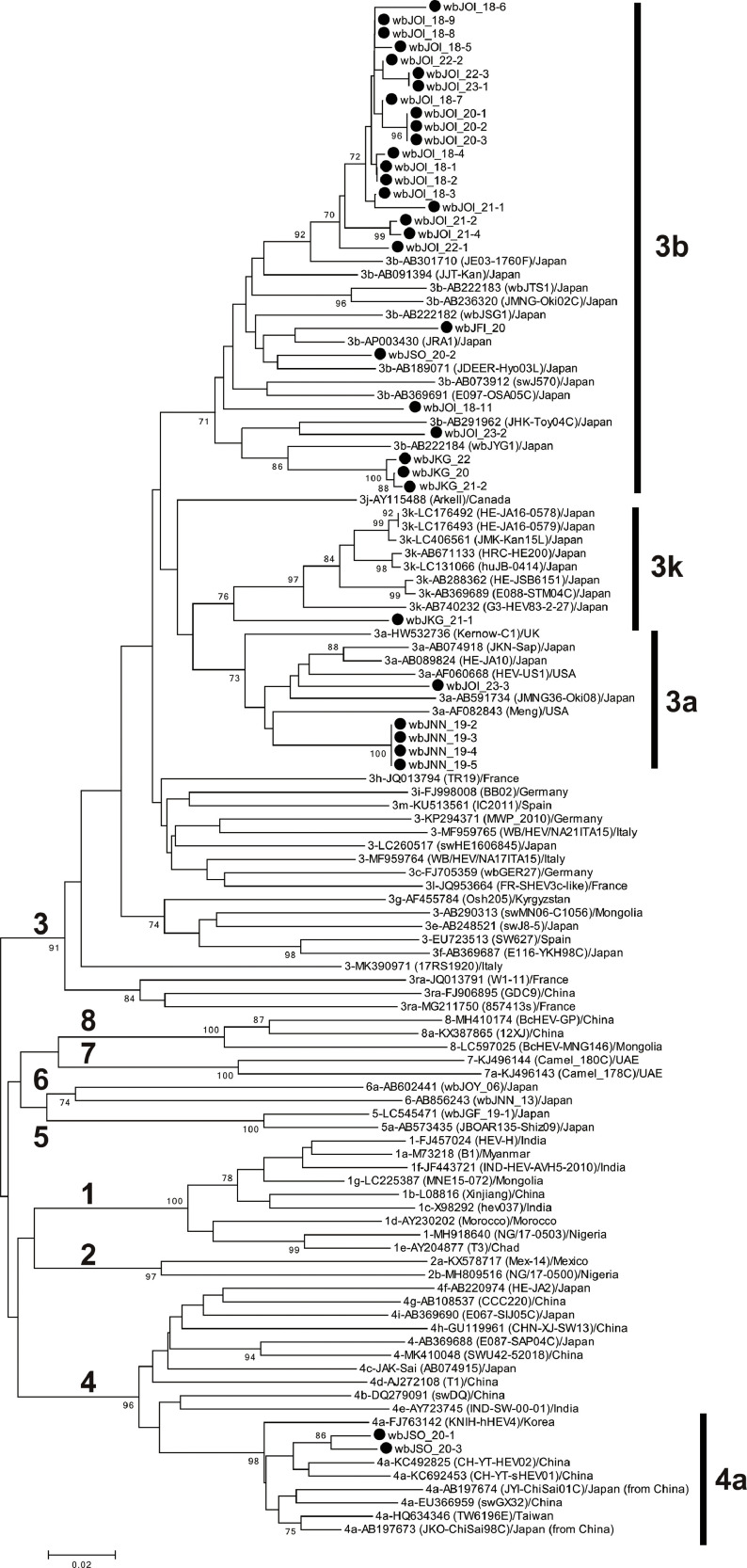
Phylogenetic tree of the 412-nt sequences within ORF2 of 81 reported reference HEV strains from HEV-1–HEV-8 as proposed by Smith et al. (2020), including additional HEV strains of subtypes 3a, 3b, 3k, and 4a, as well as HEV-5 and HEV-8, for which entire sequences have been determined, and 34 boar strains obtained in the present study; the boar strains obtained in the present study are highlighted with closed circles for clarity. The nomenclature for strain names includes abbreviations representing the region of origin: FI for Fukui, NN for Nagano, SO for Shizuoka, KG for Kagawa, and OI for Oita. Each reported HEV strain is labeled with its genotype/subtype, DDBJ/EMBL/GenBank accession number, strain name (in parentheses), and country of isolation. The phylogenetic tree was constructed using the neighbor-joining method with the Jukes-Cantor model implemented in MEGA11 (Tamura et al., 2021), with optimization of tree topology and branch lengths. The values (≥70 %) on branches represent the percentage of 1000 bootstrap replicates supporting the existence of the branches. A scale bar representing 0.02 nt substitutions per site is indicated.

**Fig. 3:**
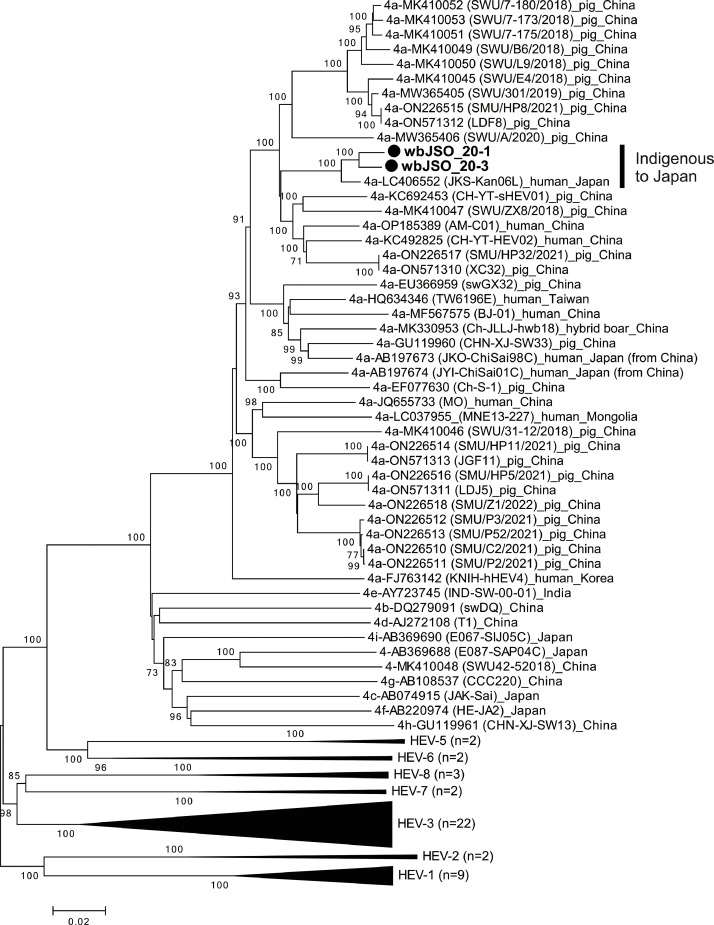
Phylogenetic tree of the entire genomic sequences of 53 reference HEV strains spanning genotypes HEV-1–HEV-8, as proposed by Smith et al. (2020), 37 additional HEV strains of subtype 4a, as well as HEV-5 and HEV-8, for which entire sequences have been determined, and two subtype 4a strains (wbJSO_20-1 and wbJSO_20-3) identified in this study, which are denoted in bold and marked with closed circles for clarity. Each reported HEV strain is annotated with its genotype/subtype, accession number from DDBJ/EMBL/GenBank, strain name (in parentheses), and country of isolation. The tree was constructed using the neighbor-joining method with the Jukes-Cantor model in MEGA11 (Tamura et al., 2021), optimized for tree topology and branch lengths. Tips are aggregated for HEV-1 (n = 9), HEV-2 (n = 2), HEV-3 (n = 22), HEV-5 (n = 2), HEV-6 (n = 2), HEV-7 (n = 2), and HEV-8 (n = 3). The values (≥70 %) on branches represent the percentage of 1000 bootstrap replicates supporting the existence of the branches. A scale bar of 0.02 nt substitutions per site is indicated.

For comparison, proposed reference sequences for subtypes of HEV (Smith et al., 2020), including additional genotype 5 and 8 HEV strains (Nishizawa et al., 2021; Takahashi et al., 2020), were used, as indicated in Fig. 2, Fig. 3 and S2.

### 2.6 Statistical analysis

Statistical analyses were performed using the Statcel 3 (OMS Publishing Inc., Saitama, Japan). The chi-squared test was used when appropriate. *P* values of <0.05 were considered to indicate significance.

## 3 Results

### 3.1 The prevalence of anti-HEV IgG antibodies and HEV RNA among wild boars

Serum and liver samples and bile suspension samples obtained from 968, 656, and 46 boars, respectively, during 2018–2023 were tested for the presence of anti-HEV IgG antibodies and/or HEV RNA (Fig. 1). Overall, 104 boars (10.7 %) were seropositive for anti-HEV IgG. The prevalence differed by geographic region (prefecture) of capture, ranging from 0 % in five prefectures to 35.0 % (7/20) in Shizuoka Prefecture. In total, 37 boars (3.6 %) had detectable HEV RNA in serum, liver, and/or bile specimens, with the prevalence differing according to the prefecture of capture, ranging from 0 % in 10 prefectures to 22.2 % (4/18) in Nagano Prefecture (Table 1). The prevalence of boar anti-HEV IgG differed markedly among the 10 prefectures with anti-HEV IgG-positive boars from 1.4 % (1/73) to 35.0 % (7/20) (median, 10.0 %). Boars with ongoing HEV infection were found in six prefectures (37.5 %), with the prevalence ranging from 2.2 % (1/46) to 22.2 % (4/18) (median, 10.2 %).

**Table 1:** Prevalence of anti-HEV IgG and HEV RNA among wild boars in Japan, during 2018–2023, stratified by geographic region.

Prefecture	No. of boars tested	Body weight: range (mean) kg []^a^	Serum			Liver		Bile		No. of HEV RNA-positive boars
			No.	Anti-HEV IgG	HEV RNA	No.	HEV RNA	No.	HEV RNA	
Ibaraki	22	20–92 (44.2) [22]	22	1 (4.5 %)	0	18	0	0		0
Tochigi	26	10–80 (44.7) [17]	26	0	0	17	0	0		0
Gunma	29	10–100 (39.8) [29]	29	0	0	29	0	0		0
Toyama	25	10–120 (36.9) [25]	25	0	0	24	0	0		0
Fukui	12	30–90 (50.0) [12]	12	1 (8.3 %)	1 (8.3 %)	12	1 (8.3 %)	0		1 (8.3 %)
Yamanashi	3	NA	3	0	0	3	0	0		0
Nagano	18	20–110 (42.6) [17]	17	2 (11.8 %)	4 (23.5 %)	17	4 (23.5 %)	0		4 (22.2 %)
Gifu	118	5–87 (34.0) [118]	118	0	0	118	0	0		0
Shizuoka	20	15–95 (39.2) [20]	20	7 (35.0 %)	3 (15.0 %)	20	3 (15.0 %)	0		3 (15.0 %)
Mie	15	13–80 (39.8) [10]	15	1 (6.7 %)	0	15	0	0		0
Hyogo	74	10–100 (42.8) [61]	73	1 (1.4 %)	0	74	0	0		0
Tottori	46	NA	0			0		46	1 (2.2 %)	1 (2.2 %)
Okayama	232	7–130 (35.0) [232]	231	5 (2.2 %)	0	232	0	0		0
Kagawa	33	12–120 (47.4) [32]	33	8 (24.2 %)	2 (6.1 %)	33	4 (12.1 %)	0		4 (12.1 %)
Oita	341	4–100 (35.3) [340]	341	77 (22.6 %)	24 (7.0 %)	41	0	0		24 (7.0 %)
Kagoshima	3	50–65 (58.3) [3]	3	1 (33.3 %)	0	3	0	0		0
Total	1017	4–130 (37.0) [938]	968	104 (10.7 %)	34 (3.5 %)	656	12 (1.8 %)	46	1 (2.2 %)	37 (3.6 %)

NA: not available.

a No. of boars whose body weight was available.

### 3.2 Genetic heterogeneity of HEV strains recovered from wild boars

HEV RNA was detectable via both ORF2/3-138 and ORF2-457 PCR in 34 boars but was detectable only by ORF2/3-138 PCR in three boars because of the low viral load. The amplification products of ORF2 (412 nt; primer sequences at both ends excluded) or ORF2/3 (98 nt) were sequenced and compared (Table 2). Thirty-four strains (91.9 %) were grouped into HEV-3 while two strains (5.4 %) were grouped into HEV-4; the remaining strain (wbJTT_20) was close to HEV-6. The phylogenetic tree constructed based on the 412-nt ORF2 sequence revealed that subtype 3b was most prevalent (n = 26, 70.3 %), followed by subtype 3a (n = 5, 13.5 %), subtype 4a (n = 2, 5.4 %), and subtype 3k (n = 1, 2.7 %) (Fig. 2, Table 2). Unlike previous surveys from 2003 to 2019 (Nishizawa et al., 2005; Sato et al., 2011; Sonoda et al., 2004; Takahashi et al., 2014; Takahashi et al., 2020; Takahashi et al., 2022), subtype 3e, subtypes 4g, and 4i were not detected, although subtype 3b remained the most prevalent (Table 2).

**Table 2:** Distribution of HEV genotype/subtype among HEV-infected boars in Japan, during 2018−2023, in comparison to that in our previous studies during 2003−2019.

Year	HEV RNA-positive boars	HEV genotype (subtype)										
		3					4				5	6
		3a	3b	3e	3k	3^a^	4a	4g	4i	4^b^		
2003−2019^c^	119	21 (17.6 %)	51 (42.9 %)	4 (3.4 %)	0	3 (2.5 %)	0	1 (0.8 %)	34 (28.6 %)	2 (1.7 %)	1 (0.8 %)	2 (1.7 %)
2018−2023^d^	37	5 (13.5 %)	26 (70.3 %)	0	1 (2.7 %)	2 (5.4 %)	2 (5.4 %)	0	0	0	0	1 (2.7%)
Total	156	26 (16.7 %)	77 (49.4 %)	4 (2.6 %)	1 (0.6 %)	5 (3.2 %)	2 (1.3 %)	1 (0.6 %)	34 (21.8 %)	2 (1.3 %)	1 (0.6 %)	3 (1.9 %)

a Although the HEV subtype could not be determined due to the low HEV load, five genotype 3 sequences were homologous to HEV subtype 3a or 3b.

b Although the HEV subtype could not be determined due to the low HEV load, two genotype 4 sequences were homologous to HEV subtype 4i.

c Retrieved from our previous studies (Sonoda et al., 2004; Nishizawa et al., 2005; Sato et al., 2011; Takahashi et al., 2014; Takahashi et al., 2020; Takahashi et al., 2022) and their HEV subtypes were determined in the present study according to the proposed reference sequences for HEV subtyping (Smith et al., 2020).

d Present study.

Since subtype 4a strains had never been obtained from domestic pigs and wild boars in Japan, their entire genomic sequences (wbJSO_20-1 and wbJSO_20-3) were determined for phylogenetic analysis with HEV-1 to HEV-8 reference strains (Smith et al., 2020) and all reported subtype 4a strains with known genomic sequences (Fig. 3). These two subtype 4a strains were grouped into a cluster formed by all reported subtype 4a strains and segregated into a subcluster, supported by a bootstrap value of 100 %, with a subtype 4a strain (JKS-Kan06L, LC406552) recovered from a Japanese patient with hepatitis E who had no history of travel abroad. The wbJSO_20-1 and wbJSO_20-3 strains were 97.5 %–97.6 % identical to the JKS-Kan06L strain over the entire genome but were only 89.1 %–93.1 % similar to the 4a strains of non-Japanese origin, including those from Japanese patients who contracted HEV infection while traveling in China (AB197673 and AB197674), suggesting that the wbJSO_20-1 and wbJSO_20-3 strains are indigenized to Japan.

Although we tried to obtain the longer sequence of the wbJTT_20 strain, which was detectable only by ORF2/3-138 PCR and close to HEV-6 within the determined 98-nt sequence, the nucleotide sequence was only extended to the 3ʹ side of the wbJTT_20 genome by 24 bases. When the nucleotide sequence identity was compared with the reported reference HEV strains within the 122-nt ORF2/ORF3-overlapping region, the wbJTT_20 strain shared 72.1 %–86.1 % identities with HEV-1–HEV-5, HEV-7, and HEV-8 and was closest to HEV-6 with an identity of 86.9 %–89.3 % (Table 3). The phylogenetic tree constructed based on the short 122-nt sequence supported the segregation HEV-6 (Supplementary Fig. S2).

**Table 3:** Comparison of the identity (%) within the partial ORF3 sequences (122 nucleotides) of the wbJTT_20 strain obtained in the present study with the previously reported reference HEV strains.

HEV strain^a^	No. of strains compared^b^	Identity (%)
HEV-1	9	75.4−78.7 (77.8 ± 1.0)
HEV-2	2	77.1 (77.1 ± 0.0)
HEV-3	22	72.1−81.2 (77.5 ± 2.0)
HEV-4	11	82.8−86.1 (84.4 ± 0.7)
HEV-5	2	82.0−84.4 (83.2 ± 1.7)
HEV-6	2	86.9−89.3 (88.1 ± 1.7)
HEV-7	2	73.8−74.6 (74.2 ± 0.6)
HEV-8	3	73.8−76.2 (74.6 ± 1.4)

a See Fig. 2 for accession numbers.

b Reference HEV strains for HEV genotype/subtype proposed by Smith et al. (2020), including additional genotype 5 and 8 HEV strains whose entire genomic sequences have been determined.

### 3.3 The decreasing prevalence of anti-HEV IgG antibodies and HEV RNA among wild boars during 2018–2023

By combining data from this study with those from our previous study conducted in 2018−2019 (Takahashi et al., 2020), we could assess the annual prevalence of HEV infection among wild boars in Japan between 2018 and 2023 (Table 4). According to data on 1453 boars, we found that the prevalence of HEV infection among wild boars tended to decrease annually, with the prevalence of anti-HEV IgG decreasing from 15.0 % (46/307) in 2018 to 8.8 % (15/170) in 2023 and that of HEV RNA decreasing from 8.3 % (26/313) in 2018 to 1.8 % (3/171) in 2023. Notably, the HEV infection among wild boars captured in 2018 and 2019 were significantly more prevalent than in those in 2022 and 2023: 14.5 % (71/488) vs. 6.2 % (30/481) for anti-HEV IgG (*P* < 0.0001) and 7.6 % (39/514) vs. 1.5 % (7/482) for HEV RNA (*P* < 0.0001).

**Table 4:** Prevalence of anti-HEV IgG and HEV RNA among wild boars in Japan, stratified by year of capture.

Year	No. of boars tested	Serum			Liver		Bile		No. of HEV RNA-positive boars [95% CI]
		No.	Anti-HEV IgG	HEV RNA	No.	HEV RNA	No.	HEV RNA	
2018	262^a^	256	29 (11.3%)	15 (5.9%)	258	15 (5.8%)	0		15 (5.7%) [3.5–9.3%]
	51	51	17 (33.3%)	11 (21.6%)	0		0		11 (21.6%) [12.4–34.9%]
	Subtotal (313)	307	46 (15.0%)	26 (8.5%)	258	15 (5.8%)	0		26 (8.3%) [5.7–12.0%]
2019	174^a^	167	23 (13.8%)	9 (5.4%)	164	8 (4.9%)	3	0	9 (5.2%) [2.6–9.7%]
	27	14	2 (14.3%)	4 (28.6%)	14	4 (28.6%)	13	0	4 (14.8%) [5.4–33.3%]
	Subtotal (201)	181	25 (13.8%)	13 (7.2%)	178	12 (6.7%)	16	0	13 (6.5%) [3.8–10.9%]
2020	199	179	24 (13.4%)	8 (4.5%)	138	5 (3.6%)	19	1 (5.3%)	9 (4.5%) [2.3–8.5%]
2021	258	243	31 (12.8%)	5 (2.1%)	165	2 (1.2%)	14	0	6 (2.3%) [1.0–5.1%]
2022	311	311	15 (4.8%)	3 (1.0%)	240	1 (0.4%)	0		4 (1.3%) [0.4–3.4%]
2023	171	170	15 (8.8%)	3 (1.8%)	99	0	0		3 (1.8%) [0.4–5.3%]
Total	1453	1391	156 (11.2%)	58 (4.2%)	1078	35 (3.2%)	49	1 (2.0%)	61 (4.2%) [3.3–5.4%]

a Retrieved from our previous report (Takahashi et al., 2020).

### 3.4 Comparison of the prevalence of anti-HEV IgG and HEV RNA in wild boars before and after the classical swine fever (CSF) outbreak in September 2018, stratified by prefecture and hunting area

Classical swine fever (CSF) is a highly contagious viral disease induced by CSF virus (CSFV), a small single-strand positive-sense RNA virus, belonging to the genus *Pestivirus* in the family *Flaviviridae* (Simmonds et al., 2017). This disease is widely distributed globally, including in Asian countries (Edwards et al., 2000). In Japan, incidents of CSF on pig farms and the isolation of CSFV from wild boars have been annually reported since a case of CSFV infection occurred on a pig farm in Gifu Prefecture within the Chubu region in September 2018 for the first time in 26 years (Isoda et al., 2020; Ito et al., 2019; Postel et al., 2019) (Supplementary Fig. S3) (MAFF, 2024a; 2024b).

As shown in Supplementary Table S1, a comparison of the changes in the positive rates of anti-HEV IgG and HEV RNA before and after the CSF outbreak (September 2018) across 16 prefectures, stratified by prefecture, revealed that the prevalence of anti-HEV IgG decreased in nine prefectures, with statistically significant reductions observed in two of these prefectures. The prevalence of HEV RNA also declined in seven prefectures, with one prefecture showing a statistically significant reduction. Between 2003 and 2023, there were 19 hunting areas where wild boars were captured both before and after the CSF outbreak within the same area. When comparing the changes in the positive rates of anti-HEV IgG and HEV RNA before and after the CSF outbreak, stratified by hunting area, the prevalence of anti-HEV IgG declined in nine areas, with statistically significant reductions observed in two of these areas (Table 5). The prevalence of HEV RNA decreased in nine areas, with two areas showing a statistically significant reduction. In addition, there were six areas where HEV RNA was not detectable both before and after the CSF outbreak.

**Table 5:** Comparison of the prevalence of anti-HEV IgG and HEV RNA in wild boars before and after the CSF outbreak in September 2018, stratified by hunting area.

Prefecture	Hunting area	Anti-HEV IgG			HEV RNA		
		Before^a^^,^^b^	After^a^^,^^c^	*P* value	Before^a^^,^^b^	After^a^^,^^c^	*P* value
Ibaraki	IK-A	4/93 (4.3%)	1/35 (2.9%)	0.7070	2/97 (2.1%)	0/35	0.3920
Tochigi	TG-A	0/8	0/16	NA	0/8	0/16	NA
Tochigi	TG-B	0/2	0/9	NA	0/5	0/9	NA
Toyama	TY-A	0/4	0/11	NA	0/4	0/11	NA
Fukui	FI-A	10/60 (16.7%)	1/17 (5.9%)	0.2620	16/61 (26.2%)	1/17 (5.9%)	0.0723
Nagano	NN-A	6/137 (4.4%)	0/29	0.2510	4/140 (2.9%)	0/30	0.3488
Nagano	NN-B	8/62 (12.9%)	2/26 (7.7%)	0.4822	1/62 (1.6%)	4/26 (15.4%)	**0.0109**
Nagano	NN-C	10/54 (18.5%)	1/33 (33.3%)	0.5268	3/54 (5.6%)	0/3	0.6749
Gifu	GF-A	74/710 (10.4%)	14/193 (7.3%)	0.1881	26/714 (3.6%)	8/193 (4.1%)	0.7438
Gifu	GF-B	5/33 (15.2%)	0/1	0.1776	5/33 (15.2%)	0/1	0.1776
Hyogo	HG-A	6/68 (8.8%)	1/108 (0.9%)	**0.0090**	0/69	0/110	NA
Tottori	TT-A	1/10 (10.0%)	0	NA	7/42 (16.7%)	1/49 (2.0%)	**0.0140**
Okayama	OY-A	3/144 (2.1%)	8/230 (3.5%)	0.4372	0/144	4/230 (1.7%)	0.1116
Okayama	OY-B	0/21	2/59 (3.4%)	0.3928	0/22	0/60	NA
Oita	OI-A	10/17 (58.8%)	40/171 (23.4%)	**0.0016**	8/17 (47.1%)	9/171 (5.3%)	**<0.0001**
Oita	OI-B	0/7	20/64 (31.3%)	0.0810	1/7 (14.3%)	4/64 (6.3%)	0.4302
Oita	OI-C	0/3	4/53 (7.5%)	0.6215	0/4	2/55 (3.6%)	0.6980
Oita	OI-D	7/28 (25.0%)	2/29 (6.9%)	0.0610	2/28 (7.1%)	0/29	0.1429
Oita	OI-E	0	1/7 (14.3%)	NA	0/3	0/8	NA
Total		144/1461 (9.9%)	97/1061 (9.1%)	0.5471	75/1514 (5.0%)	33/1117 (3.0%)	**0.0106**

NA, not applicable.

a The prevalence was compared between before and after the CSF outbreak in September 2018.

b From 2003 to August 2018.

c From September 2018 to December 2023. ^d^*P* value of <0.05 is highlighted in bold.

### 3.5 Changes in the prevalence of anti-HEV IgG and HEV RNA in wild boars following the CSF outbreak in September 2018, stratified by prefecture and hunting area

In September 2021, the Ministry of Agriculture, Forestry and Fisheries (MAFF) of Japan established standards that livestock owners must adhere to regarding hygiene management methods related to raising livestock (MAFF, 2021). The established standards mandate the installation of protective fencing for farms located in regions inhabited by wild boars. This measure aims to prevent wild boars from entering sanitary control areas of farms.

When comparing the changes in the positive rates of anti-HEV IgG and HEV RNA before (September 2018–2021) and after (2022–2023) the implementation of livestock hygiene management standards by the MAFF of Japan in September 2021 across 16 prefectures, stratified by prefecture, the prevalence of anti-HEV IgG decreased in seven prefectures, with statistically significant reductions observed in two (Gifu and Okayama) (Supplementary Table S2). The prevalence of HEV RNA also declined in five prefectures, with one prefecture (Gifu) showing a statistically significant reduction. Between September 2018 and 2023, there were 15 hunting areas both before and after the implementation of livestock hygiene management standards where wild boars were captured within the same area. A comparison of the changes in the positive rates of anti-HEV IgG and HEV RNA before and after the implementation of livestock hygiene management standards by the MAFF of Japan (September 2021), stratified by hunting area, revealed that the prevalence of anti-HEV IgG declined in 11 areas, with statistically significant reductions observed in two (GF-A in Gifu and OY-A in Okayama) (Table 6). The prevalence of HEV RNA decreased in six areas, with one area (GF-A in Gifu) showing a statistically significant reduction. Notably, HEV RNA was undetectable in 12 of 15 areas in 2022–2023. Furthermore, eight areas had no detectable HEV RNA both before and after the implementation of livestock hygiene management standards, indicating the persistence of no HEV infection within these areas. Overall, in the 15 hunting areas, the prevalence of anti-HEV IgG statistically significantly declined from 12.8 % (76/594) to 6.7 % (30/451) (*P* = 0.0011), and HEV RNA also statistically significantly declined from 4.2 % (25/598) to 1.5 % (7/452) (*P* = 0.0140) (Table 6).

**Table 6:** Changes in the prevalence of anti-HEV IgG and HEV RNA in wild boars by hunting area following the CSF outbreak in September 2018.

Prefecture	Hunting area	Anti-HEV IgG			HEV RNA		
		2018–2021^a^	2022–2023^a^	*P* value	2018–2021^a^	2022–2023^a^	*P* value
Ibaraki	IK-A	1/34 (2.9%)	0/1	0.8619	0/34	0/1	NA
Gunma	GM-A	0/1	0/18	NA	0/1	0/18	NA
Toyama	TY-A	0/6	0/5	NA	0/6	0/5	NA
Fukui	FI-A	1/14 (7.1%)	0/3	0.6333	1/14 (7.1%)	0/3	0.6333
Gifu	GF-A	14/93 (15.1%)	0/100	**<0.0001**^b^	8/93 (8.6%)	0/100	**0.0027**
Hyogo	HG-A	1/88 (1.1%)	0/20	0.6320	0/89	0/21	NA
Okayama	OY-A	8/150 (5.3%)	0/80	**0.0355**	4/150 (2.7%)	0/80	0.1406
Okayama	OY-B	1/6 (16.7%)	1/53 (1.9%)	0.0579	0/7	0/53	NA
Okayama	OY-C	1/1 (100%)	0/3	0.2500	0/1	0/3	NA
Kagawa	KG-A	10/30 (33.3%)	1/13 (7.7%)	0.0768	3/30 (10.0%)	1/13 (7.7%)	0.8109
Oita	OI-A	24/83 (28.9%)	16/88 (18.2%)	0.0975	6/83 (7.2%)	3/88 (3.4%)	0.2636
Oita	OI-B	9/31 (29.0%)	11/33 (33.3%)	0.7106	1/31 (3.2%)	3/33 (9.1%)	0.3327
Oita	OI-C	3/41 (7.3%)	1/12 (8.3%)	0.9067	2/43 (4.7%)	0/12	0.4466
Oita	OI-D	2/13 (15.4%)	0/16	0.1039	0/13	0/16	NA
Oita	OI-F	1/3 (33.3%)	0/6	0.1336	0/3	0/6	NA
Total		76/594 (12.8%)	30/451 (6.7%)	**0.0011**	25/598 (4.2%)	7/452 (1.5%)	**0.0140**

NA, not applicable.

a The prevalence was compared before (September 2018–2021) and after (2022–2023) the implementation of livestock hygiene management standards by the MAFF of Japan in September 2021.

b *P* value of <0.05 is highlighted in bold.

## 4 Discussion

HEV infection among wild boars has been a subject of concern because of the zoonotic potential and the public health implications (Fanelli et al., 2021; Kim et al., 2011; Li et al., 2005; Matsuda et al., 2003; Okano et al., 2014; Rivero-Juarez et al., 2017; Tamada et al., 2004; Wang et al., 2019). The present study on 1017 wild boars across 16 Japanese prefectures between 2018 and 2023 revealed a 10.7 % seroprevalence of anti-HEV IgG antibodies and a 3.6 % prevalence of HEV RNA. Geographic variations were notable, with some regions showing no seropositive cases and others, like Shizuoka Prefecture, displaying high seroprevalence rates. HEV RNA was detected in six prefectures, with the highest prevalence in Nagano Prefecture. Genetic analysis of HEV strains found in these boars identified primarily HEV-3 strains, with subtypes 3b and 3a being the most common. Notably, the presence of HEV-4a strains, which have not been previously detected in domestic pigs and wild boars in Japan, suggests potential indigenization of these strains. Over the five-year period, there was a significant decline in HEV infection rates among wild boars, with anti-HEV IgG prevalence dropping from 15.0 % in 2018 to 8.8 % in 2023, and HEV RNA prevalence decreasing from 8.3 % to 1.8 % over the same period. This decrease was more pronounced in certain prefectures and hunting areas, especially following the CSF outbreak in 2018 and the implementation of livestock hygiene management standards by the MAFF of Japan in 2021. Statistical analysis confirmed significant reductions in HEV infection rates post-CSF outbreak and post-hygiene management standards implementation, indicating effective control measures.

The present study demonstrated considerable variability in the prevalence of HEV infection among wild boars in Japan across different regions (Table 1) and periods (Table 4). The observed variability in the prevalence of HEV infection among wild boars could have several explanations. First, environmental or ecological factors may have affected wild boar populations or their interactions with other animal species, including small- and medium-sized animals such as rats, foxes, raccoon dogs, and bats, which can easily enter pig farms and impact HEV transmission dynamics (Oi and Nagai, 2024). Second, external factors, such as emerging infectious diseases, may have influenced the prevalence of HEV in wild boars. As described below in detail, CSFV infection, which occurred in 2018 for the first time in 26 years in Japan, may be a plausible contributing factor to the observed changes in HEV infection among wild boars.

The genetic diversity observed among HEV strains recovered from wild boars offers valuable insights into the molecular epidemiology of HEV infection in Japan. Data obtained during 2018–2023 showed that most strains were grouped into HEV-3 (91.9 %), with subtype 3b being the most prevalent, followed by subtype 3a. Furthermore, two strains (5.4 %) were grouped into HEV-4, while one strain (wbJTT_20) closely resembled HEV-6. Notably, subtype 4a strains are prevalent in China (Li et al., 2022; Zhu et al., 2011) and have not been previously obtained from domestic pigs or wild boars in Japan. However, two subtype 4a strains identified in this study, wbJSO_20-1 and wbJSO_20-3, exhibited close genetic similarity (>97 % over the entire genome) to a subtype 4a strain (JKS-Kan06L, LC406552) recovered from a Japanese patient with hepatitis E without a history of foreign travel, suggesting their indigenous origin in Japan. These findings underscore the ongoing evolutionary dynamics and transmission patterns of HEV.

The identification of subtype 4a HEV strains closely related to those found in human cases in Japan raises concerns about potential zoonotic transmission, emphasizing the need for enhanced surveillance and control measures. In addition, subtype 3k HEV strains have been identified from humans and from a domestic pig (AB740232) in Japan (Miura et al., 2017; Shiota et al., 2013). The indigenous presence of a subtype 3k strain in Japan is supported by the detection of this strain from wild boar in the present study. Furthermore, although limited by the low viral load and potential RNA degradation in bile suspension, a novel HEV-6 (wbJTT_20) was identified that was closely related to two previously reported HEV-6 strains (Takahashi et al., 2014; Takahashi et al., 2011), albeit with a lower identity range of 86.9 %–89.3 %. Remarkably, during the preparation of this manuscript, another novel HEV-6 strain (wbJHG_23) exhibiting a high similarity of 98.4 % was identified from a wild boar captured in Hyogo Prefecture, adjacent to Tottori Prefecture where wbJTT_20 was found (Primadharsini et al., 2024). Thus far, HEV-6 strains have not been detected in domestic pigs, suggesting their exclusive circulation among wild boars in Japan, albeit with lower frequency.

The recent declining trend in the prevalence of HEV infection among wild boars in Japan identified herein prompted our inquiry into the contributing factors driving this phenomenon. Consequently, we explored the potential role of CSFV infection in wild boars as a determinant of the decline in HEV infection. CSFV can infect both domestic pigs and wild boars, with transmission occurring bidirectional (Brugh et al., 1964; Fritzemeier et al., 2000; Fukai et al., 2023). Notably, wild boars can act as reservoirs for CSFV infection in domestic pigs because of the highly contagious nature of this virus (Meng et al., 2009). Sporadic cases of CSFV infection have persisted, as exemplified by an instance at a pig farm in the Chubu region in September 2018 (Postel et al., 2019), followed by annual reports of CSF cases at pig farms and isolation of CSFV from wild boars (MAFF, 2024a, 2024b). Notably, an analysis at the prefectural level revealed that before the CSF outbreak, the prevalence of anti-HEV IgG in wild boars varied across 16 prefectures. Post-outbreak data indicated a decrease in anti-HEV IgG prevalence in nine prefectures, with statistically significant reductions in two of these (Supplementary Table S1). Similarly, HEV RNA prevalence declined in seven prefectures, with one showing a statistically significant reduction, indicating a lower rate of active HEV infections post-outbreak.

Geographically, Japan is characterized by mountainous regions, which may cause the behavior of boar colonies and the HEV prevalence rate to vary from ridge to ridge. Indeed, it has been reported that HEV prevalence differed by hunting area even within the same prefecture (Motoya et al., 2016). Consequently, a hunting area-level analysis was conducted (Table 5). Among the 19 hunting areas where wild boars were captured both before and after the CSF outbreak, nine areas exhibited a decline in anti-HEV IgG prevalence, with significant reductions in two. HEV RNA prevalence also decreased in nine areas, with two showing significant reductions. Notably, six areas had no detectable HEV RNA both before and after the outbreak, suggesting no new HEV infections in these regions. This stability in the absence of HEV RNA might indicate the presence of effective natural barriers or limited HEV circulation in these areas.

Given that a certain level of HEV infiltration among boars had already been established prior to the CSF outbreak, the efficacy of strengthening CSF prevention measures to reduce contact between farm pigs and wild boars might have been limited. In September 2021, the MAFF of Japan established standards for livestock owners regarding hygiene management methods related to raising livestock (MAFF, 2021). These standards mandate the installation of protective fences on farms located in wild boar habitat areas to prevent wild boars from entering sanitary control areas of farms. Furthermore, regular inspections of the protective fence and other equipment for damage, with immediate repair when necessary, are required (MAFF, 2020). Consequently, CSF prevention measures aimed at reducing contact between farm pigs and wild boars likely became more effective following the implementation of these livestock hygiene management standards by the MAFF of Japan in September 2021.

Analysis at the prefectural level post-implementation of these standards revealed a decrease in the prevalence of anti-HEV IgG in seven prefectures and a decline in HEV RNA prevalence in five prefectures, with a significant reduction in Gifu, where the first CSF outbreak was recorded in September 2018 (Supplementary Table S2). These findings suggest that the hygiene standards may have been effective in reducing HEV exposure and transmission among wild boars, likely due to improved management of livestock-wildlife interfaces. Further analysis at the hunting area level between September 2018 and 2023 across 15 hunting areas showed that 11 areas experienced a decline in anti-HEV IgG prevalence, and six areas showed a decrease in HEV RNA prevalence (Table 6). Notably, one area (GF-A in Gifu) exhibited a significant reduction in HEV RNA. Remarkably, HEV RNA was undetectable in 12 of the 15 areas during 2022–2023, indicating a substantial reduction in active HEV infections. Furthermore, eight areas had no detectable HEV RNA both before and after the implementation of the hygiene standards, suggesting a persistent absence of HEV infection in these regions. However, the presence of infected boars in the remaining three areas (KG-A in Kagawa and OI-A and OI-B in Oita) might be attributed to regional disparities in the implementation and seriousness of these measures, depending on the local infection situation (Bungo-Ohno Livestock Hygiene Center, 2020).

Overall, in the 15 hunting areas, the prevalence of anti-HEV IgG significantly declined from 12.8 % to 6.7 %, and HEV RNA prevalence significantly declined from 4.2 % to 1.5 %. These statistically significant reductions underscore the potential impact of the CSF outbreak and the subsequent livestock hygiene management standards on reducing HEV prevalence in wild boar populations.

In conclusion, this study highlights a significant reduction in HEV infection rates among wild boars in Japan over a five-year period, with notable geographic variations. The decline is linked to the CSF outbreak in 2018 and the implementation of livestock hygiene management standards by the MAFF of Japan in 2021, which reduced contact between farm pigs and wild boars. Statistical analysis confirmed significant reductions in HEV prevalence among wild boars following the CSF outbreak and the adoption of hygiene standards. However, the number of hunting areas where a statistically significant decrease was observed were limited due to the small numbers of boars in each studied area. Further investigation is warranted to explore the association between countermeasures against CSFV infection and the decrease in HEV infection among a larger number of wild boars across more hunting areas. In addition, it is necessary to continue monitoring for new HEV infections in areas where HEV infection is currently not observed and to access whether HEV prevalence will decrease in areas where HEV infection is currently present.

## Funding

This work was supported in part by grants from the Research Program on Hepatitis from the Japan Agency for Medical Research and Development, AMED (to H.O.: JP22fk0210075, JP24fk0210132).

## CRediT authorship contribution statement

**Masaharu Takahashi:** Investigation, Formal analysis, Data curation, Conceptualization. **Tsutomu Nishizawa:** Visualization, Investigation, Data curation. **Akira Nishizono:** Resources, Conceptualization. **Manri Kawakami:** Resources, Conceptualization. **Yukihiro Sato:** Resources. **Kazunori Kawakami:** Resources. **Masahiko Irokawa:** Resources. **Tomoko Tamaru:** Resources. **Shinichi Miyazaki:** Resources. **Mizuho Shimada:** Resources. **Hideaki Ozaki:** Resources. **Putu Prathiwi Primadharsini:** Investigation. **Shigeo Nagashima:** Investigation. **Kazumoto Murata:** Supervision. **Hiroaki Okamoto:** Writing – review & editing, Writing – original draft, Visualization, Supervision, Project administration, Funding acquisition, Data curation, Conceptualization.

## Declaration of competing interest

The authors declare that they have no known competing financial interests or personal relationships that could have appeared to influence the work reported in this paper.

## Data Availability

Data will be made available on request.

## Appendix Supplementary materials

Image, application 1
