# Supplementary material for: Recent decline in hepatitis E virus prevalence among wild boars in Japan: Probably due to countermeasures implemented in response to outbreaks of classical swine fever virus infection
Source: Virus Res. 2024 Jul 19;348:199438. doi: 10.1016/j.virusres.2024.199438 (PMC11315222; doi:10.1016/j.virusres.2024.199438)
Supplement: Supplementary file 1 [file mmc1.pdf]

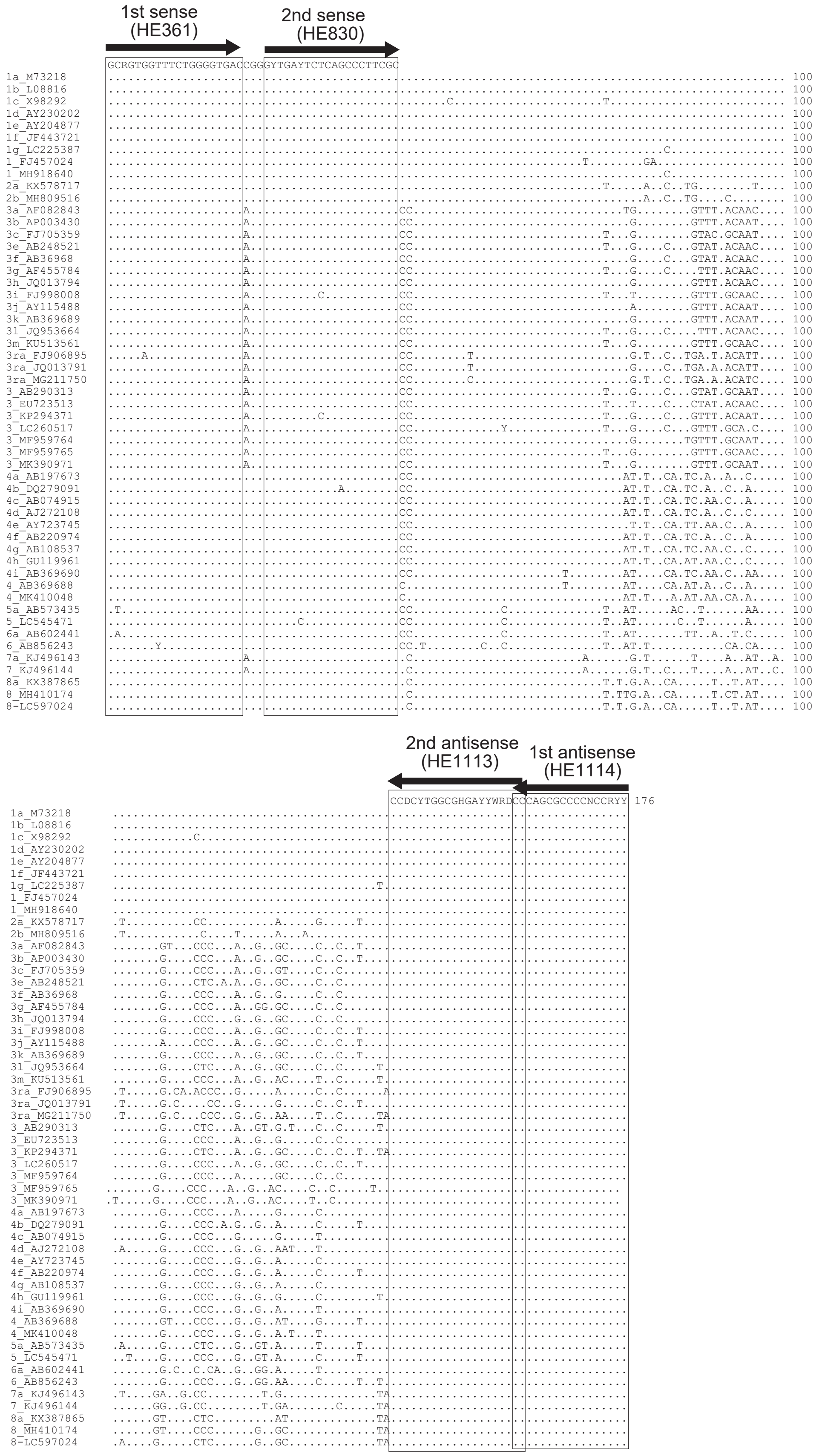

Supplementary Fig. S1 Nucleotide sequences and positions of primers for ORF2/3-138 RT-PCR. A comprehensive comparison of 53 HEV reference sequences, spanning genotypes HEV-1 to HEV-8 (Smith et al., 2020), conducted for four primer sequences: HE361 (sense primer for 1st round PCR), HE1114 (antisense primer for cDNA synthesis and 1st round PCR), HE830 (sense primer for 2nd round PCR), and HE1113 (antisense primer for 2nd round PCR). In this figure, the following nucleotide symbols are used: R (A/G), Y (T/C), N (G/A/T/C), W (A/T), H (A/T/C), and D (G/A/T). Identical nucleotides to those at the top are indicated by dots.

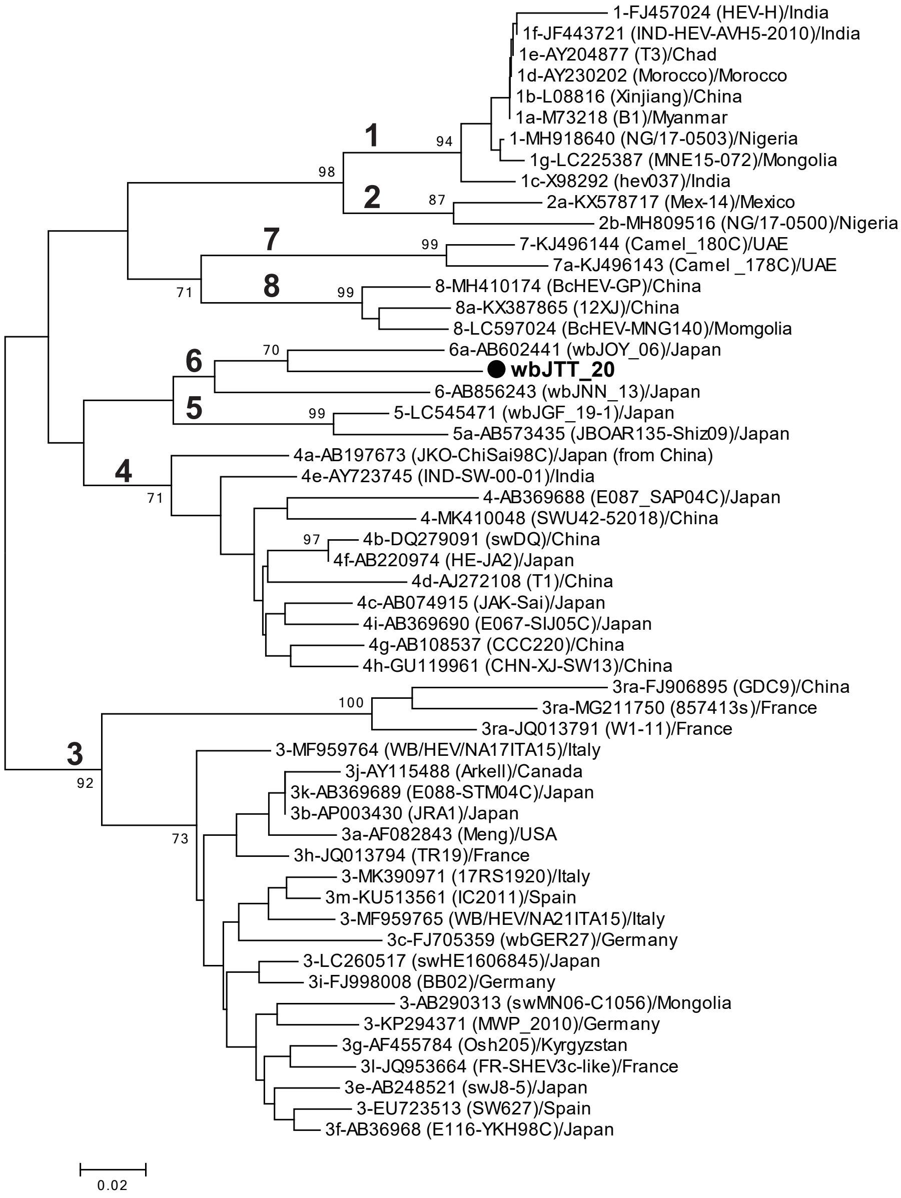

Supplementary Fig. S2. Phylogenetic tree of the 122-nt sequences within the ORF2/ORF3 overlapping region of 53 reported reference HEV strains belonging to HEV-1–HEV-8 as proposed by Smith et al. (2020), including additional HEV strains of HEV-5 and HEV-8, for which entire sequences have been determined, and wbJTT_20 strain obtained in the present study. The boar strains obtained in this study are indicated in bold and highlighted with closed circles for clarity. Each reported HEV strain is labeled with its genotype/subtype, DDBJ/EMBL/GenBank accession number, strain name (in parentheses), and country of isolation. The phylogenetic tree was constructed using the neighbor-joining method with the Jukes-Cantor model implemented in MEGA11 (Tamura et al., 2021), with the optimization of tree topology and branch lengths. The values (≥70%) on branches represent the percentage of 1000 bootstrap replicates supporting the existence of the branches. A scale bar representing 0.02 nt substitutions per site is indicated.

A

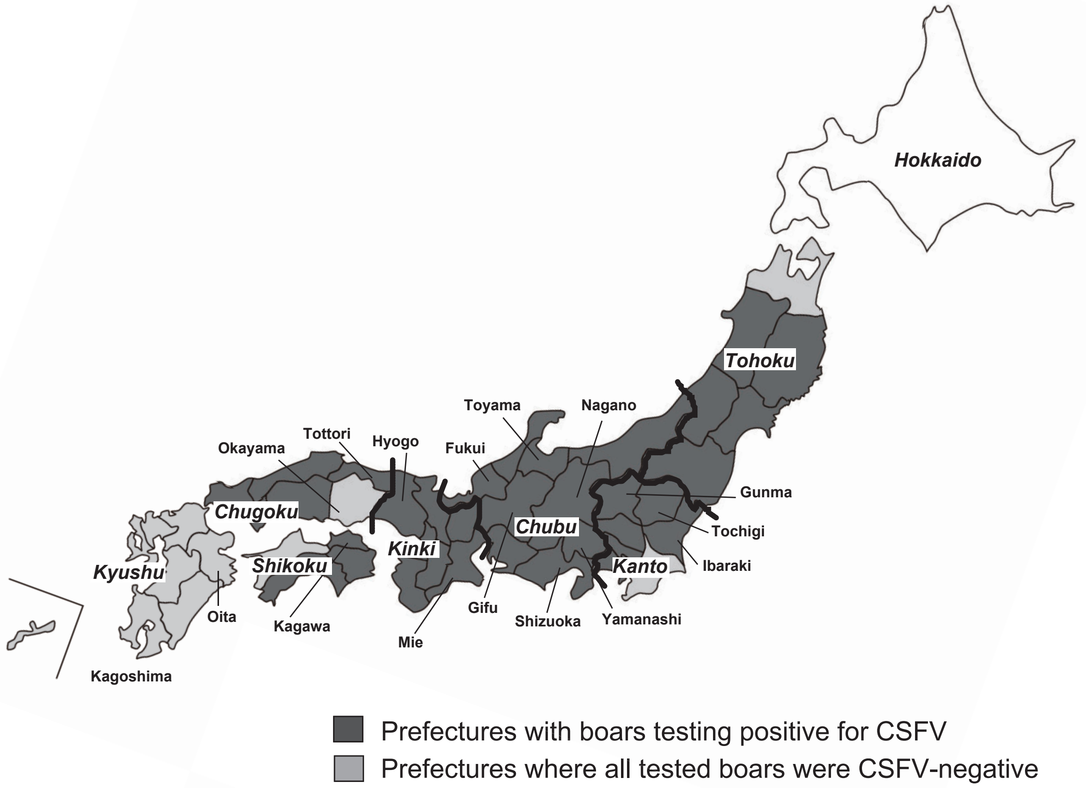

B

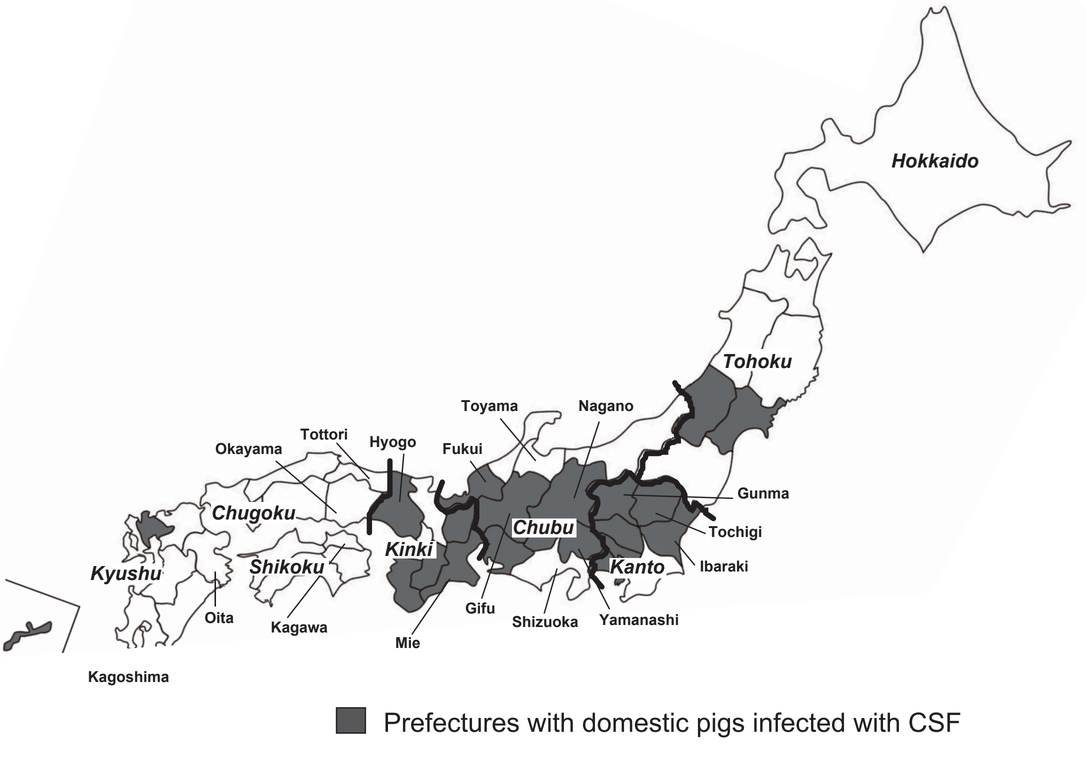

Supplementary Fig. S3. A map delineating the prefectures of Japan where CSFV-positive wild boars were detected (A) and those where domestic pigs afflicted with CSF (B) were discovered from September 2018 to December 2023. Prefectures where wild boars were captured in the present study are indicated for reference. Data were retrieved from the MAFF of Japan (MAFF, 2024a, 2024b).

Supplementary Table S1

Comparison of the prevalence of anti-HEV IgG and HEV RNA in wild boars before and after the CSF outbreak in September 2018, stratified by prefecture.

Prefecture	No. of boars	No. of hunting area ( )[a]	Anti-HEV IgG			HEV RNA		
			Before[b,c]	After[b,d]	*P* value	Before[b,c]	After[b,d]	*P* value
Ibaraki	188	9 (1)	5/147 (3.4%)	1/35 (2.9%)	0.8713	4/153 (2.6%)	0/35	0.3336
Tochigi	314	8 (2)	2/285 (0.7%)	0/26	0.6683	0/288	0/26	NA
Gunma	29	3 (0)	0	0/29	NA	0	0/29	NA
Toyama	52	6 (1)	0/22	0/25	NA	0/27	0/25	NA
Fukui	82	4 (1)	11/63 (17.5%)	1/17 (5.9%)	0.2355	16/65 (24.6%)	1/17 (5.9%)	0.0898
Yamanashi	11	2 (0)	0	0/3	NA	2/8 (25.0%)	0/3	0.3384
Nagano	366	11(3)	27/282 (9.6%)	5/65 (7.7%)	0.6364	9/300 (3.0%)	5/66 (7.6%)	0.0793
Gifu	1014	4 (2)	84/815 (10.3%)	14/194 (7.2%)	0.1914	31/820 (3.8%)	8/194 (4.1%)	0.8231
Shizuoka	28	4 (0)	2/2 (100%)	7/20 (35.0%)	0.0746	2/8 (25.0%)	3/20 (15.0%)	0.5325
Mie	15	1 (0)	0	1/15 (6.7%)	NA	0	0/15	NA
Hyogo	179	1 (1)	6/68 (8.8%)	1/108 (0.9%)	**0.0090**[e]	0/69	0/110	NA
Tottori	121	4 (1)	1/37 (2.7%)	0	NA	7/72 (9.7%)	1/49 (2.0%)	0.0951
Okayama*	862	13 (2)	37/492 (7.5%)	11/294 (3.7%)	**0.0323**	15/567 (2.6%)	4/295 (1.4%)	0.2211
Kagawa	82	2 (0)	5/39 (12.8%)	11/43 (25.6%)	0.1453	0/39	4/43 (9.3%)	0.0508
Oita*	414	10 (5)	17/69 (24.6%)	69/338 (20.4%)	0.4335	11/73 (15.1%)	16/341 (4.7%)	**0.0011**
Kagoshima*	10	1 (0)	0	2/10 (20.0%)	NA	0	0/10	NA
With CSF[f]	2481	59 (12)	143/1760 (8.1%)	41/580 (7.1%)	0.4125	71/1849 (3.8%)	22/632 (3.5%)	0.6818
Without CSF (*)[g]	1286	24 (7)	54/561 (9.6%)	82/642 (12.8%)	0.0855	26/640 (4.1%)	20/646 (3.1%)	0.3507
Total	3767	83 (19)	197/2321 (8.5%)	123/1222 (10.1%)	0.1194	97/2489 (3.9%)	42/1278 (3.3%)	0.3465

NA, not applicable.

[a] No of hunting areas where wild boars were captured both before and after the CSF outbreak in September 2018, within the same hunting area.

[b] The prevalence was compared between before and after the CSF outbreak in September 2018.

[c] From 2003 to August 2018.

[d] From September 2018 to December 2023.

[e] *P* value of <0.05 is highlighted in bold.

[f] Prefecture where classical swine fever virus (CSFV) infection in pigs or wild boars was detected by December 2023.

[g] Prefectures where CSFV infection in pigs and/or wild boars had not been confirmed by December 2023.

Supplementary Table S2

Changes in the prevalence of anti-HEV IgG and HEV RNA in wild boars by prefecture following the CSF outbreak in September 2018.

Prefecture	No. of boars	No. of hunting areas ()[a]	Anti-HEV IgG			HEV RNA		
			2018–2021[b]	2022–2023[b]	*P* value	2018–2021[b]	2022–2023[b]	*P* value
Ibaraki	35	1 (1)	1/34 (2.9%)	0/1	0.8619	0/34	0/1	NA
Tochigi	26	3 (0)	0/9	0/17	NA	0/9	0/17	NA
Gunma	29	3 (1)	0/1	0/28	NA	0/1	0/28	NA
Toyama	25	3 (1)	0/20	0/5	NA	0/20	0/5	NA
Fukui	17	1 (1)	1/14 (7.1%)	0/3	0.6333	1/14 (7.1%)	0/3	0.6333
Yamanashi	3	1 (0)	0	0/3	NA	0	0/3	NA
Nagano	66	4 (0)	5/65 (7.7%)	0	NA	5/66 (7.6%)	0	NA
Gifu	194	2 (1)	14/94 (14.9%)	0/100	**<0.0001**[c]	8/94 (8.5%)	0/100	**0.0029**
Shizuoka	20	3 (0)	7/20 (35.0%)	0	NA	3/20 (15.0%)	0	NA
Mie	15	1 (0)	1/15 (6.7%)	0	NA	0/15	0	NA
Hyogo	110	1 (1)	1/88 (1.1%)	0/20	0.6320	0/89	0/21	NA
Tottori	49	1 (0)	0	0	NA	1/49 (2.0%)	0	NA
Okayama*	295	4 (3)	10/158 (6.3%)	1/136 (0.7%)	**0.0117**	4/159 (2.5%)	0/136	0.0626
Kagawa	43	1 (1)	10/30 (33.3%)	1/13 (7.7%)	0.0768	3/30 (10.0%)	1/13 (7.7%)	0.8109
Oita*	341	8 (5)	41/183 (22.4%)	28/155 (18.1%)	0.3240	10/186 (5.4%)	6/155 (3.9%)	0.5128
Kagoshima*	10	1 (0)	2/10 (20.0%)	0	NA	0/10	0	NA
With CSF[d]	632	25 (7)	40/390 (10.3%)	1/190 (0.5%)	**<0.0001**	21/441 (4.8%)	1/191 (0.5%)	**0.0076**
Without CSF (*)[e]	646	13 (8)	53/351 (15.1%)	29/291 (10.0%)	0.0524	14/355 (3.9%)	6/291 (2.1%)	0.1695
Total	1278	38 (15)	93/741 (12.6%)	30/481 (6.2%)	**0.0003**	35/796 (4.4%)	7/482 (1.5%)	**0.0042**

NA, not applicable.

[a] No. of hunting areas where wild boars were captured both before (September 2018–2021) and after (2022–2023) the implementation of livestock hygiene management standards by the MAFF of Japan in September 2021, within the same hunting area.

[b] The prevalence was compared between the periods September 2018–2021 and 2022–2023.

[c] *P* value of <0.05 is highlighted in bold.

[d] Prefecture where classical swine fever virus (CSFV) infection in pigs or wild boars was detected by December 2023.

[e] Prefectures where CSFV infection in pigs and/or wild boars had not been confirmed by December 2023.
